# Formation of polymorphs and pores in small nanocrystalline iron oxide particles

**DOI:** 10.1038/s41598-022-19276-4

**Published:** 2022-09-12

**Authors:** Alexander Levish, Markus Winterer

**Affiliations:** Nanoparticle Process Technology, Department of Mechanical and Process Engineering, University of Duisburg-Essen, Lotharstr. 1, 47057 Duisburg, Germany

**Keywords:** Chemical engineering, Condensed-matter physics, Nanoscale materials, Techniques and instrumentation, Nanoscale materials, Techniques and instrumentation

## Abstract

A novel chemical vapor synthesis reactor design is used to control the pore-particle mesostructure and investigate the pore formation mechanism through the variation of residence time in oxygen. This enables the exploitation of the Kirkendall effect at the nanoscale to generate ultrasmall pores in small nanocrystalline iron oxide particles. Detailed structural characterization and quantitative data analysis of complementary high resolution transmission electron microscopy images, X-ray diffractograms, nitrogen sorption isotherms and X-ray absorption spectra provide a consistent comprehensive picture of the hollow nanoparticles from the local to the microstructure. The pore formation mechanism seems to play a key role for β-Fe_2_O_3_ polymorph formation.

## Introduction

Iron (III) oxide, Fe_2_O_3_ is a polymorphic material^1^ with the phase depending on pressure, temperature and size^2^. Discovered in 1956, β-Fe_2_O_3_ is reported to be a metastable intermediate form of Fe_2_O_3_ with the Bixbyite crystal structure^3^. Since its discovery it has been obtained by different processes such as chemical vapor condensation^4^, spray pyrolysis^5^, impregnation of iron compounds into a mesoporous matrix^6^ or via atomic layer deposition^7^.

As metastable phase β-Fe_2_O_3_ can be stabilized for example by contributions to the free energy through interfaces via film-substrate-interaction in epitaxy^7^ or surfaces in case of particles. This may be described for spherical particles by Eq. (1) and is particularly relevant for nanoscaled materials^1^:

1 $$\frac{G}{V} = \frac{\eta }{\upsilon } + \frac{6\gamma }{d}$$

where *G* is the free energy, *V* the volume, *η* the chemical potential, *υ* the molar volume, *γ* the surface enthalpy and *d* the particle diameter. Suresh et al.^8^ derived an estimate for the dependence of the phase transition temperature *T*_*t*_ on particle diameter by adding a surface (or interface) enthalpy term to Gibbs free energy:

2 $$T_{t} = T_{t\infty } \left( {1 + 6\frac{{\Delta_{t} \gamma }}{{\Delta_{t} h}} \cdot \frac{1}{d}} \right)$$

where *T*_*t∞*_ is the phase transition temperature in the bulk, *∆*_*t*_*γ* is the difference of the surface (or interface) enthalpy and *∆*_*t*_*h* the enthalpy of transformation per volume for the phases considered. Additionally, surface stress may play an important role^9^. This size dependence is especially relevant in the regime of small nanoparticles (below about 10 nm to 20 nm) since a significant fraction of atoms is located at the particle surface and may lead to the formation or stabilization of polymorphs considered unstable or metastable in bulk form. Therefore, size (respectively curvature) may be used as parameter to control the phase composition^2^. The formation of a specific polymorph like β-Fe_2_O_3_ may also depend on (i) slight variations in composition through non-stoichiometry, (ii) doping or surface chemistry for example by water adsorption or OH-group termination (through *∆*_*t*_*γ* in Eq. 2), (iii) the path of formation for example from metallic iron, cementite, oxide or hydroxide particles or (iv) kinetic stabilization of high temperature phases by quenching.

**Figure 1:**
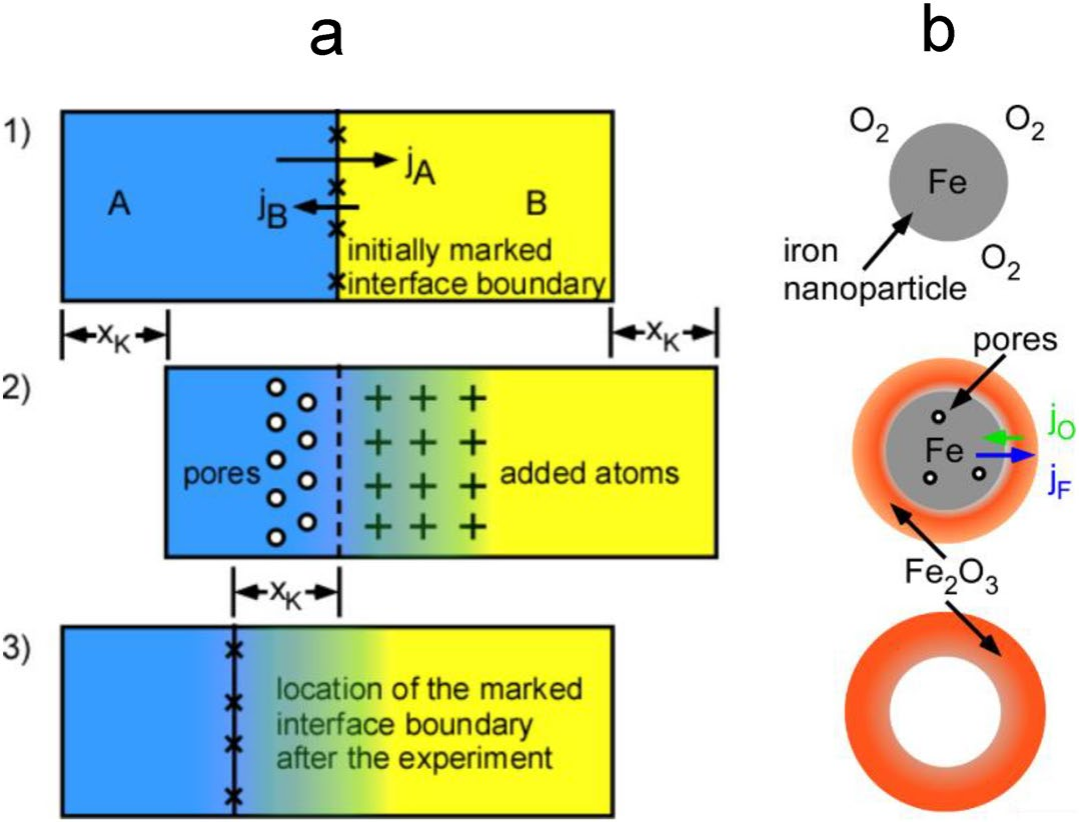
Kirkendall effect in case of bulk systems (**a**)^17^ and nano-particles (**b**).

Hollow particles containing intraparticular pores can be generated in gas phase synthesis for example by spray or reactive drying pyrolysis^10^ and the Kirkendall effect^11^. During spraying a molecular precursor solution or a nanoparticle dispersion ‘egg’-shell type particles may form when droplets are dried and decomposed through precipitation of solutes or particles at the liquid–vapor interface. Lee et al. proposed this as mechanism for the formation of Fe_2_O_3_ hollow spheres from iron acetylacetonate containing droplets^4^. In mechanisms based on the Kirkendall effect hollow particles are formed from existing solid, dense particles^11^. The Kirkendall effect describes the movement of interfaces in solids caused by different atomic diffusion coefficients for the different elements comprising the material^12^. The bulk Kirkendall effect is visualized in Fig. 1a. Initially, (Fig. 1a(1)) two metals with different diffusion coefficients are in contact. Upon heat treatment of the diffusion couple due to unequal diffusion of the different metal atoms (Fig. 1a(2)) vacancies and later pores form on the side of the fast-diffusing metal (A) and adatoms or added material on the side of the slower diffusing metal (B) where an alloy is formed.

Since material A diffses faster into material B, the interface is shifted into the direction of the material with the faster diffusion coefficient (Fig. 1a(3)). In oxides generated from metals via oxidation the Kirkendall effect may be observed when the diffusion coefficients of metal and oxygen atoms are different in the oxide film generated by reaction at the gas–solid interface. The Kirkendall effect is also observed at the nanoscale and may even be used for generation of hollow structures in particles. A schematic illustration is shown in the Fig. 1b. In this case an oxide is formed as shell on a metallic core. An ultrasmall pore is formed in the nanoparticles when the metal ion diffuses faster into the oxide shell than the oxygen anions into the metal core. Vacancy clusters are formed and grow into pores with a hollow oxide particle as end product as shown for example for hollow ZnO nanoparticles generated from Zn metal nanoparticles^11^.

Particles containing cavities or pores which modify the materials properties extrinsically are advantageous in different applications also depending on the material system, i.e. on their intrinsic properties. Bonil et al. show recently the beneficial use of hollow iron oxide structures in battery applications, where the structure improves the lithium intercalation and consequently the capacity^13^. Ma et al. observe increased performance of chemical sensors for hematite multiwall hollow particles made by a hydrothermal method^14^. Hollow encapsulated iron containing nanoparticles can be used as active catalyst in hydrogen production^14^. In medicine, hollow spheres may be used for transport of special compounds^15^ including their controlled release or uptake. β-Fe_2_O_3_ itself is also of interest in the field of electro catalysis^16^ and photoassisted water oxidation^7^.

One way to generate nanoscaled iron oxides with controlled phase composition is chemical vapor synthesis (CVS) known for synthesis of pure nanoparticles of high crystallinity^18^. Different phases including polymorphs of Fe_2_O_3_^19^ can be produced in nanoparticles by varying synthesis parameters^20^. Besides synthesis temperature and reactant partial pressures residence time is an important CVS process parameter^21^ which can be well controlled in a novel CVS setup to produce phase pure β-Fe_2_O_3_ nanoparticles as described in this contribution. In CVS different processes operate in parallel, for example coagulation, coalescence and in our case additionally the Kirkendall effect for the formation of hollow particles. If the temperature is too low, few or very small particles are formed, if it is too high, particles coalesce completely. In preliminary experiments, optimal parameter windows for the formation of hollow spheres regarding temperature and pressure had been identified and are kept constant in the study presented here where we use residence time as independent variable to study the temporal evolution of hollow particles and phases.

Here, we present results regarding the impact of residence time in oxygen on the formation of ultrasmall pores in small nanocrystalline Fe_2_O_3_ particles, elucidate the nanoscale Kirkendall effect as pore formation mechanism and characterize nanocrystalline β-Fe_2_O_3_ regarding local, crystal and microstructure.

## Experimental methods

### Synthesis of hollow Fe_2_O_3_ nanoparticles

**Figure 2:**
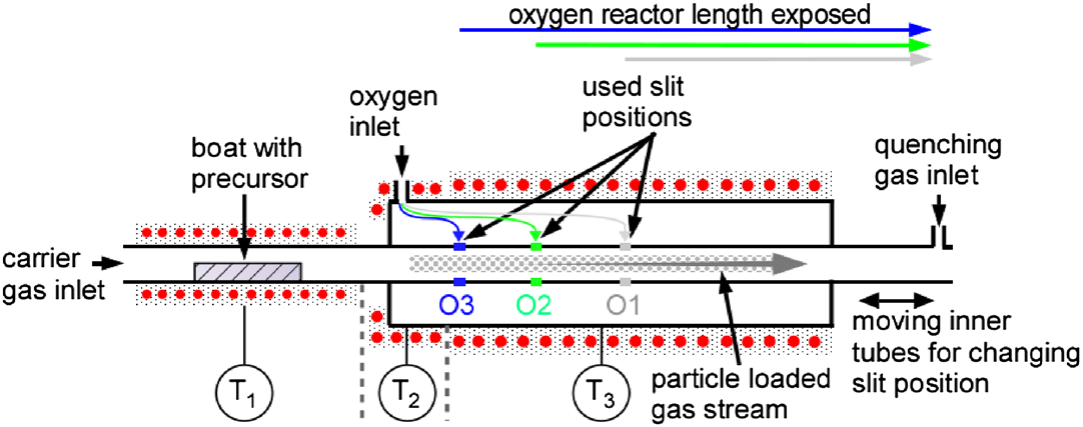
CVS reactor using two concentric tubes and associated temperature regions. Slit positions are marked with different colors and are assigned to the sample O1 (grey), O2 (green), O3 (blue) with increasing oxidation time. Above the reactor proposed particle formation and transformation steps are assigned.

A novel CVS reactor is designed and constructed. It allows to exploit the nanoscale Kirkendall effect generating a specific morphology and microstructure in nanoparticles and to study their evolution, The Kirkendall effect depends on temperature and time and the materials system through solid state diffusion. In the system described here by reaction of metallic iron particles with oxygen. Accordingly, the CVS setup is modified to enable the variation of the reaction time in oxygen, τ_ox_, by adjusting the inlet position for oxygen into the hot wall reactor. The reaction zone consists of a double tube placed concentric in a hot wall furnace according to Fig. 2. The outer tube (35 mm outer and 29 mm inner diameter) is connected to the preheated reaction gas, oxygen. The inner tube (24 mm outer and 18 mm inner diameter) is positioned in line and concentric with the outer tube separated 2 mm from each other and divided in two parts which can be moved continuously in direction of the reactor axis. This way a cylindrical slit allows gas to flow between outer and inner tube at defined positions followed by isothermal mixing. The slit positions where the gas from the outer tube enters the reaction zone are displayed in Fig. 2. Four positions (Table 1): (i) R3: ∞ mm, 0 ms; (ii) O1: 350 mm, 285 ms; (iii) O2: 150 mm, 399 ms; (iv) O3: 0 mm, 484 ms are chosen to learn about (i) the initial state before oxidation, (ii) emerging pores and (iii and iv) conversion by growth of pores, coalescence and phase transformation with significant, observable difference regarding particle characteristics.

**Table 1:** Sample notation with relevant variable synthesis parameters.

Sample notation	Estimated oxidation time, τ_ox_ (ms)	Position of the oxygen gas inlet downstream the reaction zone (mm)
R3	0	∞
O1	285	350
O2	399	150
O3	484	0

Iron acetylacetonate, Fe(acac)_3_, is thermally evaporated at 180 °C in a ceramic boat (T_1_ in Fig. 2) and brought to the required temperature by means of a heating jacket. An average mass flow of the precursor of 12.5 mg/min is observed. A helium flow of 1 slm is introduced at the carrier gas inlet and 2 slm of preheated (250 °C, T_2_ in Fig. 2) oxygen is fed into the outer tube. The hot wall temperature of the reactor is set to 800 °C (T_3_ in Fig. 2). After the reaction zone nitrogen at ambient temperature (2 slm) is introduced into the system to quench the aerosol (nanoparticle gas mixture) temperature. The system pressure is held constant at 400 mbar and the product powder (nanoparticles) is collected thermophoretically downstream. The major advantage of this coaxial design is the avoidance of cold spots and isothermal addition of reaction gas, since oxygen and particle-loaded gas stream have approximately the same temperature at the point of mixing.

The precursor vapor is converted into nanoparticles by CVS. Precursor decomposition occurs via pyrolysis in case of late oxygen introduction and oxidation otherwise. In general, initially metallic iron nanoparticles in a carbonaceous matrix are formed. Those are then converted to nanoparticles with a growing oxide shell and pore as they proceed through the reactor. Overall, the chemical reactions may be summarized as

$$2{\text{Fe}}({\text{C}}_{5} {\rm H}_{7} {\rm O}_{2} )_{3} \xrightarrow{{\Delta Q}}2{\text{Fe}} + 18{\text{C}} + 12{\text{CO}} + 21{\rm H}_{2} \xrightarrow{{42{\rm O}_{2} }}{\text{Fe}}_{2} {\rm O}_{3} + 30{\text{CO}}_{2} + 21{\rm H}_{2} {\rm O}$$

By variation of τ_ox_ it is possible to investigate the evolution of the hollow nanoparticles. The further downstream the slit is placed, the less time nanoparticles are exposed to oxygen. The samples presented here are named as follows (Table 1): O1 for nanoparticles with a τ_ox_ of 285 ms (position marked in grey in Fig. 2) corresponding to a slit position 350 mm downstream of the start of the reaction zone and the shortest τ_ox_, O2 for nanoparticles with a τ_ox_ of 399 ms (position marked in green in Fig. 2) corresponds to a slit position 150 mm downstream of the start of the reaction zone and O3 for nanoparticles with a τ_ox_ of 484 ms (position marked in blue in Fig. 2) corresponds to a slit positioned at the start of the reaction zone and thus τ_ox_ is equal to the maximum residence time in the reactor. Sample R3 is synthesized similar to sample O3, but without oxygen, instead nitrogen is added to the system at the corresponding slit position. Sample R3 serves as a reference for an oxygen-free synthesis.

### Characterization

Morphology (shape) and microstructure, i. e. size (distribution) and arrangement of crystallites, pores and shell are characterized by transmission electron microscopy (HRTEM, JEOL JEM 2200 FS). Overall, more than 1650 nanoparticles and more than 500 pores are inspected in micrographs and counted for the quantitative analysis. Nitrogen sorption (Quantachrome Autosorb 1C) is measured at − 196 °C and analyzed using the Brunauer, Emmett and Teller (BET) method to obtain the specific surface area (S) and density functional theory (DFT) to compute the pore size distribution. Prior to the sorption measurement samples are outgassed in vacuum for 24 h at 250 °C. X-ray diffraction (XRD) measurements are performed using an X-Pert Pro powder diffractometer in the range of 15°–100° 2θ of dry as-synthesized powders. X-ray diffractograms are analyzed by Rietveld refinement through MAUD to determine phase content and crystal structure^22^. Crystallographic data from literature are used as starting point for the refinement: β-Fe_2_O_3_ (ICSD number: 237290)^23^, γ-Fe_2_O_3_ (ICSD number: 87121)^24^ and α-Fe_2_O_3_ (ICSD number: 15840)^25^. X-ray absorption spectroscopy (XAFS) measurements are performed on sample O2 in transmission at the Fe-K-edge using a Si (111) monochromator and ionization chambers as detectors at beamline P65 at PETRA III at DESY (Hamburg, Germany). Sample absorption is optimized by homogeneous dilution in starch. The energy scale of all spectra is calibrated relative to the value for the Fe-K-edge using a simultaneously measured metallic α-iron foil (the first maximum of the derivate of the spectrum is at 7112 eV)^26^. Additionally, commercial reference materials are measured: α- and γ-Fe_2_O_3_ (Sigma-Aldrich Chemie GmbH, iron (III) oxide and iron (III) oxide nanopowder). The FEFF9 code^27^ is used to compute XANES as well as amplitude and phase functions for EXAFS.

XAFS data are quantitatively analyzed regarding XANES (X-ray Absorption Near Edge Structure) using empirical line shapes with the xafsX code^28^ and using Reverse Monte Carlo simulations (RMC) in the EXAFS (Extended X-ray Absorption Fine Structure) region with the rmcxas code^29^. RMC analysis is performed using initial atomic configurations generated from the crystallographic results of the Rietveld refinement of the XRD data of sample O2.

## Results

### Electron microscopy, nitrogen sorption and X-ray diffraction

**Figure 3:**
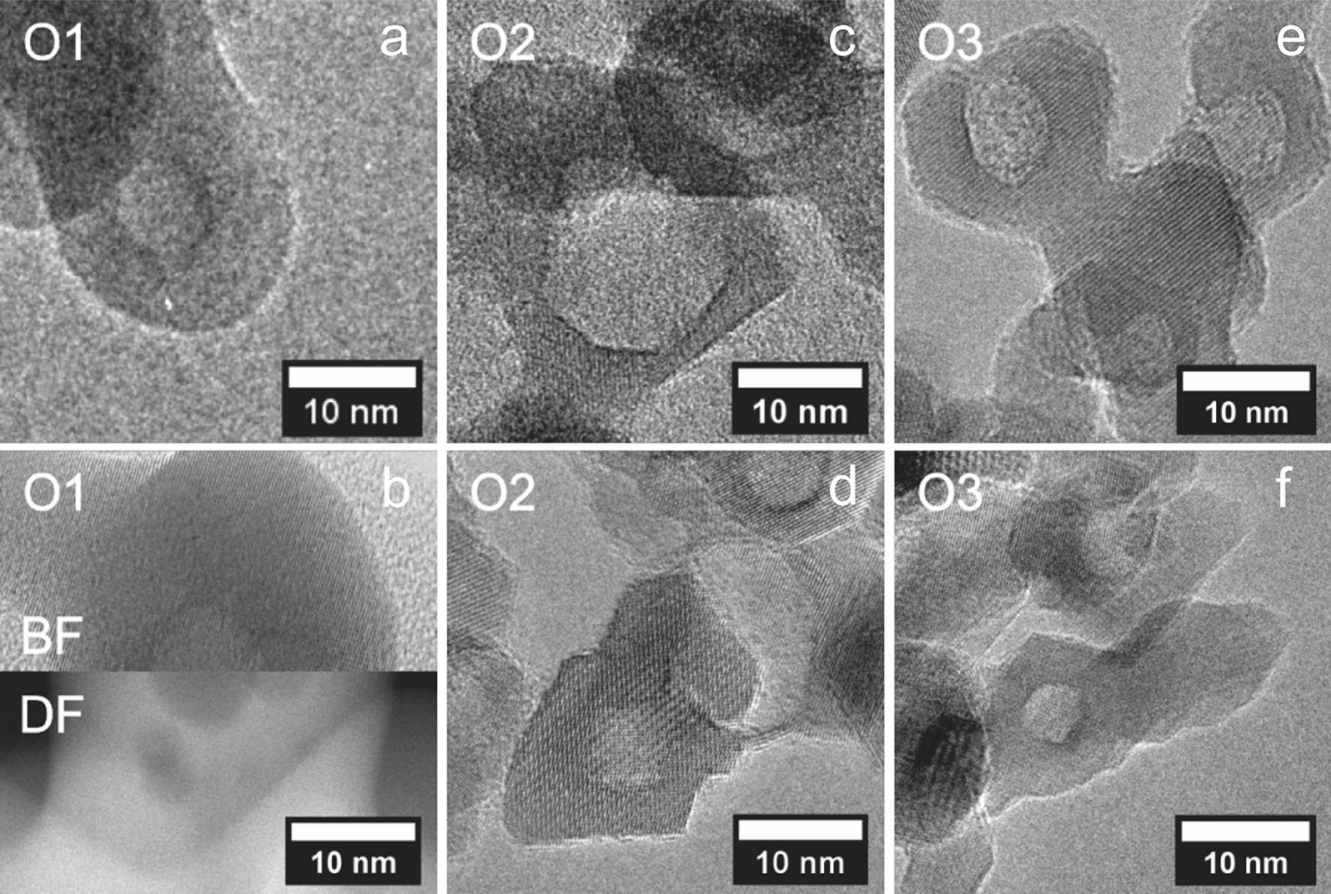
Single nanoparticle images obtained from powder samples. TEM images for sample O1 (**a**), combined DF-BF STEM image for sample O1 with multiple pores within a single particle (**b**), sample O2 (**c**–**d**) and sample O3 (**e**–**f**).

Nanoparticles synthesized without oxygen (sample R3) result in ultrasmall carbon coated metallic nanoparticles similar to those presented in our previous work^30^. This indicates that in the absence of oxygen, the carbon contained in the precursor ligands is pyrolyzed and deposited on the metallic nuclei thereby preventing further growth and oxidation^30^. All oxidic samples (O1 to O3) contain small (poly-) crystalline nanoparticles with even smaller pores as observed in representative enlarged HRTEM micrographs (Fig. 3). O1 consists of smaller nanoparticles with emerging pores (Fig. 3a), the nanoparticles may contain also more than one pore. Those can be observed precisely by combining dark-field (DF) and bright-field (BF) STEM imagining (Fig. 3b). In samples O2 and O3 we observe pores similar to those discovered by Lee et al.^4^. The pores are mostly not spherical but isometric and facetted.

**Figure 4:**
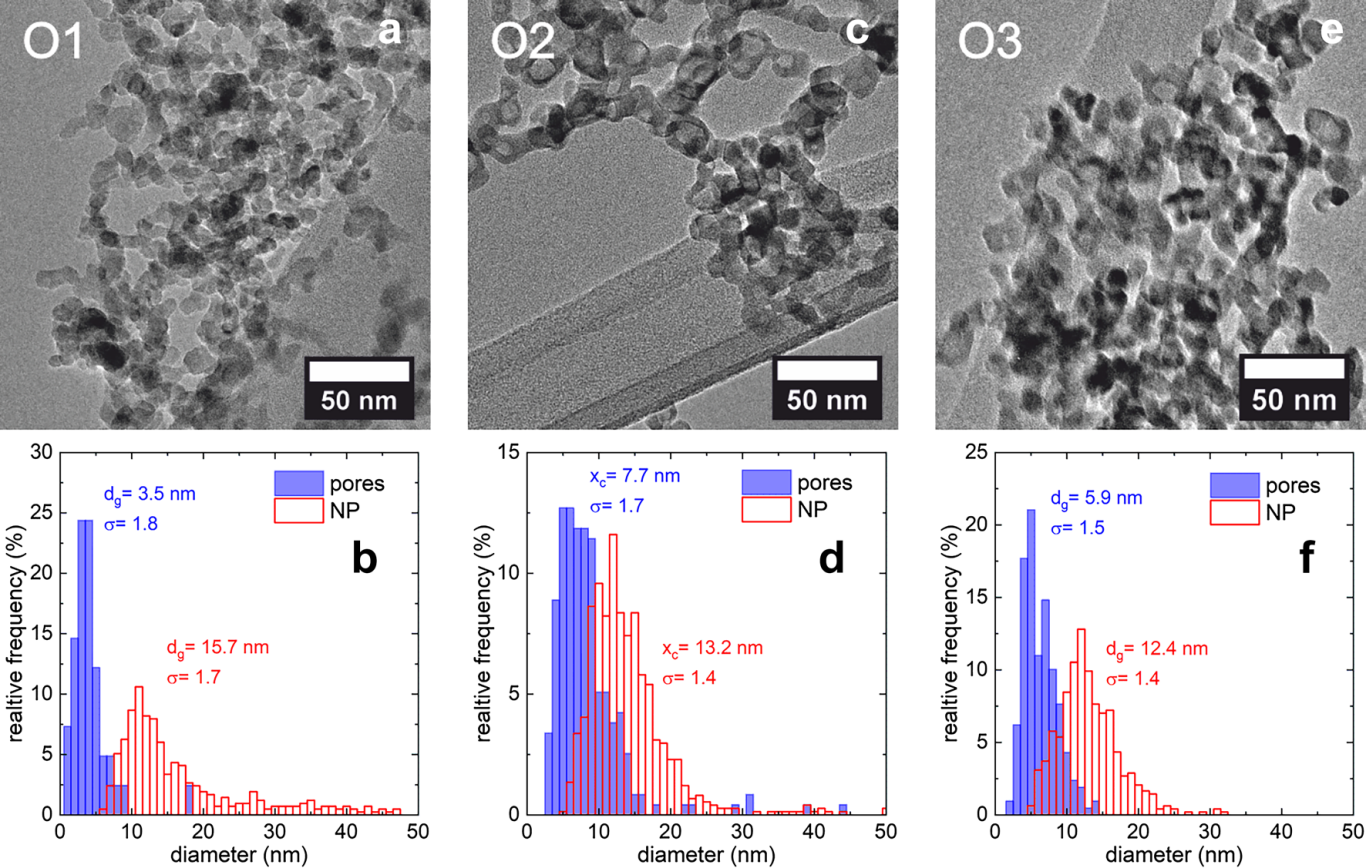
Representative TEM micrographs for samples O1 (**a**), O2 (**c**), O3 (**e**) and corresponding size distributions of the nanoparticles and pores below, O1 (**b**), O2 (**d**) and O3 (**f**).

Agglomerates including sintering necks between the nanoparticles are observed in all batches (Fig. 4a,c,e). The majority of nanoparticles in samples O2 and O3 contain one pore per particle. A lower fraction of hollow particles is observed in sample O1. However, as the pores in sample O1 are just emerging they may be very small and difficult to detect.

Histograms of the size distributions together with numerically determined geometric mean and geometric standard deviations values are shown in Fig. 4b,d,f. The mean nanoparticle diameter obtained from the image analysis does decrease only slightly with slit position or τ_ox_ respectively (Fig. 4b,d,f). This is due to the similar CVS conditions for the initial particle formation step in the process. However, O1 contains the smallest pores, O2 the largest and with further increase of τ_ox_ in O3 the pore diameter is shrinking again.

**Figure 5:**
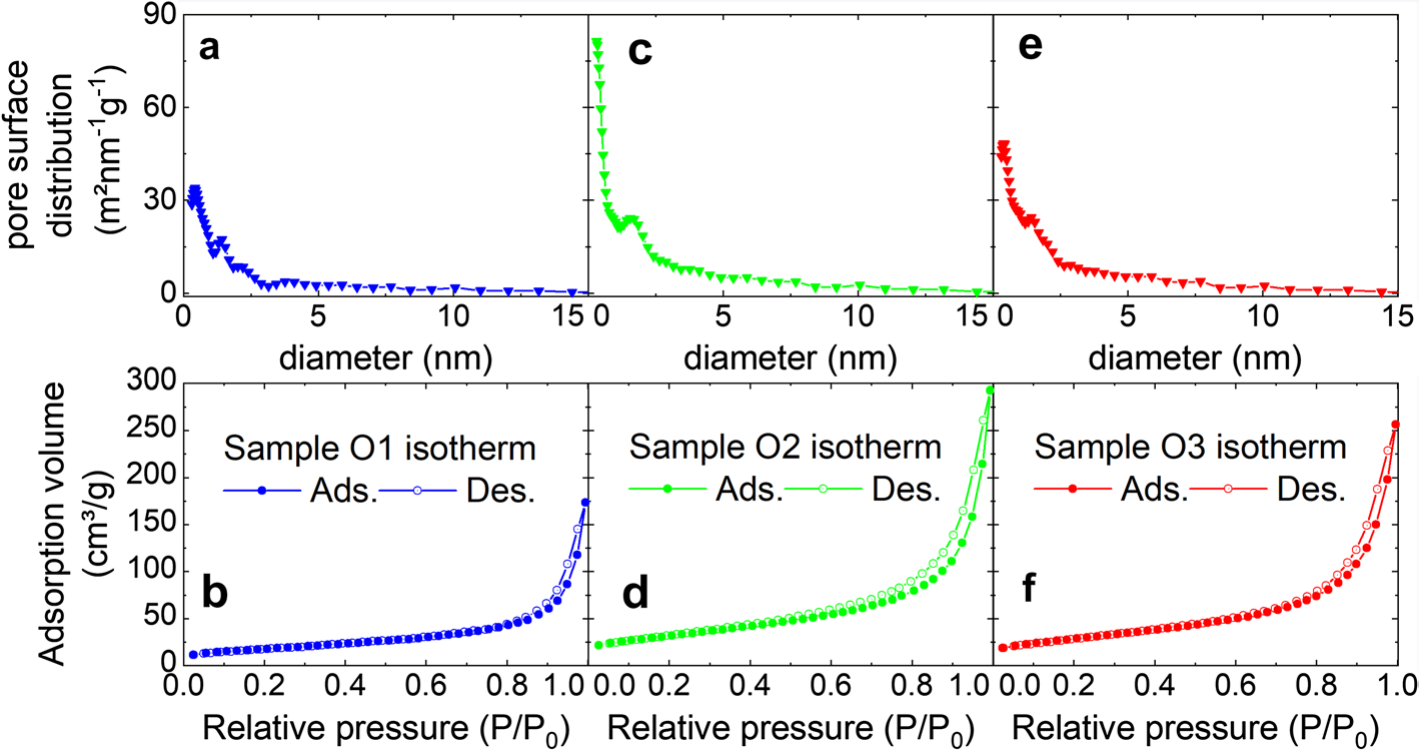
Nitrogen adsorption isotherms for Samples O1 (**b**), O2 (**d**) and O3 (**f**) with the corresponding DFT pore surface distribution (**a**,**c**,**e**).

Complete nitrogen adsorption and desorption isotherms are measured for samples O1 to O3 (Fig. 5). The specific surface areas as determined from analysis of the BET isotherm. The largest specific surface area is observed for the intermediate oxidation time in sample O2 (116.2 m^2^/g). Samples O3 and O1 have lower specific surface areas of 102.4 m^2^/g and 63.5 m^2^/g. Correspondingly, the particle size d_BET_ determined from these values assuming monodisperse, dense, and spherical particles has a minimum for O2. The isotherms for all samples are similar and displayed in Fig. 5 together with the DFT pore surface distribution. The isotherms can be referred to as type III isotherm. They contain narrow hysteresis of type H4 or H3 above p/p_0_ = 0.6. An exact assignment is difficult due to their narrowness. These results indicate that the particles contain both mesopores and micropores that are slit-like^31^. Accordingly, DFT pore size distributions in Fig. 5a,c,e show micropores (< 2 nm) and mesopores (> 2 nm). The distributions are at least bimodal for all samples with the highest microporosity in sample O2.

**Figure 6:**
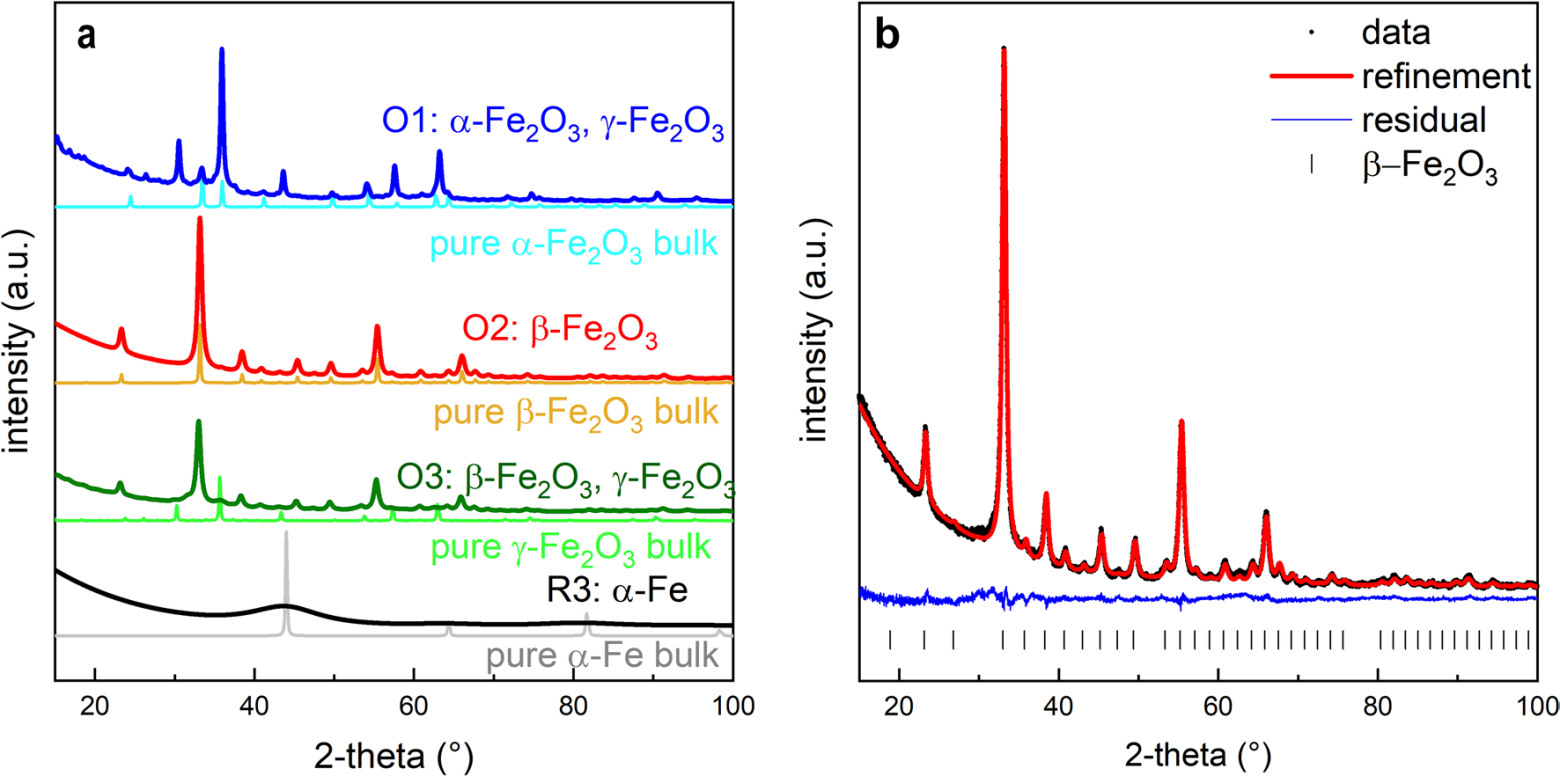
X-ray diffraction pattern of samples O1, O2, O3 and R3 (**a**), additionally diffraction pattern of corresponding pure phases are shown with only instrumental broadening included. The refined dataset for the O2 is displayed in (**b**) with residual signal and β-Fe_2_O_3_ reflex positions.

X-ray powder diffraction provides information about phase content, crystal- and microstructure (crystallite size and microstrain) through Rietveld refinement. All samples are crystalline regarding the iron containing phases, even sample R3 with very small cluster like nanoparticles as shown by the existing if very broad Bragg reflections (Fig. 6a). Table 2 summarizes the XRD results regarding phase content, crystal and micro-structure. Sample R3 consists of ultrasmall metallic iron nanocrystals in a carbon matrix which are similar to the metallic nanoparticles studied previously (see Enz et al. for a detailed discussion^30^). However, when oxygen is added during synthesis, the carbon matrix is destroyed, allowing coalescence, growth and facilitating oxidation of the metallic nanoparticles. According to XRD, samples O1, O2, O3 are completely converted into crystalline Fe_2_O_3_ nanoparticles. In sample O1 γ-Fe_2_O_3_ (87 wt.%) and α-Fe_2_O_3_ (13 wt.%) phases are observed with crystallite sizes of about 30 nm. Sample O2 consists of pure β-Fe_2_O_3_ with a crystallite size of about 20 nm, results of the Rietveld refinement are displayed in Fig. 6b. Sample O3 experienced the longest oxidation time and contains 62 wt.% of β-Fe_2_O_3_ with a crystallite size of about 20 nm and the remainder consists of 10 nm γ-Fe_2_O_3_ nanocrystals.

**Table 2:** Content of the crystalline phase, crystallographic, microstructural information obtained by Rietveld refinement of X-ray diffractograms and crystallographic densities of corresponding phase in the samples.

Sample	Phase		Lattice parameters (Å)	Crystallite size (nm)	Microstrain (%)	Density (g/cm^3^)
	Content	wt%				
R3	α-Fe	100 (–)	a = 2.91 (–)	1.1 (6)	2.2 (3)	7.536
O1	α-Fe_2_O_3_	12.9 (1)	a = 5.04 (–) b = 13.76 (1)	32 (2)	0.13 (3)	5.266
	γ-Fe_2_O_3_	87.1 (1)	a = 8.34 (–) c = 25.05 (–)	29.2 (3)	0.05 (2)	4.857
O2	β-Fe_2_O_3_	100 (–)	a = 9.40 (–)	19.9 (1)	0.16 (3)	5.105
O3	β-Fe_2_O_3_	60.1 (6)	a = 9.40 (–)	19.7 (1)	0.21 (6)	5.104
	γ-Fe_2_O_3_	39.9 (1)	a = 8.22 (–) c = 26.62 (2)	9.3 (2)	0.4 (4)	4.844

A comparison of the size parameters determined from TEM, BET and XRD results can be found in Table 3. Analysis of XRD, TEM and nitrogen adsorption data reveal intriguingly different results regarding particle and pore size. In order to explain these differences, one has to remember that these characterization methods used ‘observe’ different features as particle and ‘measure’ size with different weights. In XRD analyzed by Rietveld refinement the volume weighted coherent diffracting domain size d_XRD_ (here shortly called crystallite size) is measured whereas d_BET_ measures the size of monodisperse dense spheres equivalent to the specific surface area, i. e. is surface weighted. And finally image analysis of TEM micrographs results in number weighted size distributions for particles and pores which allows the derivation of higher weighted diameters comparable to XRD (d_3_ for d_XRD_) and BET (d_2_ for d_BET_) results. This provides a detailed picture of particle morphology and hierarchical microstructure. Since the volume weighted diameter from TEM images (d_3_) is larger than the crystallite size d_XRD_ we conclude that the shell in the porous particles is polycrystalline. The smaller particle size obtained from BET d_BET_ compared to the surface weighted diameter from TEM images (d_2_) points to a significantly large fraction of pores in samples O1 to O3.

**Table 3:** Mean crystallite size (column length from Rietveld refinement of XRD), nanoparticle and pore diameter distributions determined from TEM images and results of numerical analysis of distributions, geometric mean and geometric standard deviation together with surface, d_2_, and volume weighted diameter, d_3_, and specific surface area S measured by BET and particle size.

Sample	XRD	TEM						BET	
	Crystal	Particle				Pore		Particle	
	d_XRD_ (nm)	d_g_ (nm)	σ (–)	d_2_ (nm)	d_3_ (nm)	d_g_ (nm)	σ (–)	S (m^2^/g)	d_BET_ (nm)
R3	1 (1)	1.6 (4)	–	–	–	–	–	–	–
O1	30 (2)	15.7 (1)	1.7 (2)	41 (2)	54 (2)	3.5 (2)	1.8 (1)	63.5 (1.2)	19.1 (4)
O2	20 (0.1)	13.2 (1)	1.4 (1)	23 (2)	32 (2)	7.7 (1)	1.7 (1)	116.2 (2.0)	10.1 (2)
O3	16 (0.2)	12.4 (1)	1.4 (1)	16 (1)	18 (1)	5.9 (1)	1.5 (1)	102.4 (2.0)	11.7 (2)

### X-ray absorption spectroscopy

**Figure 7:**
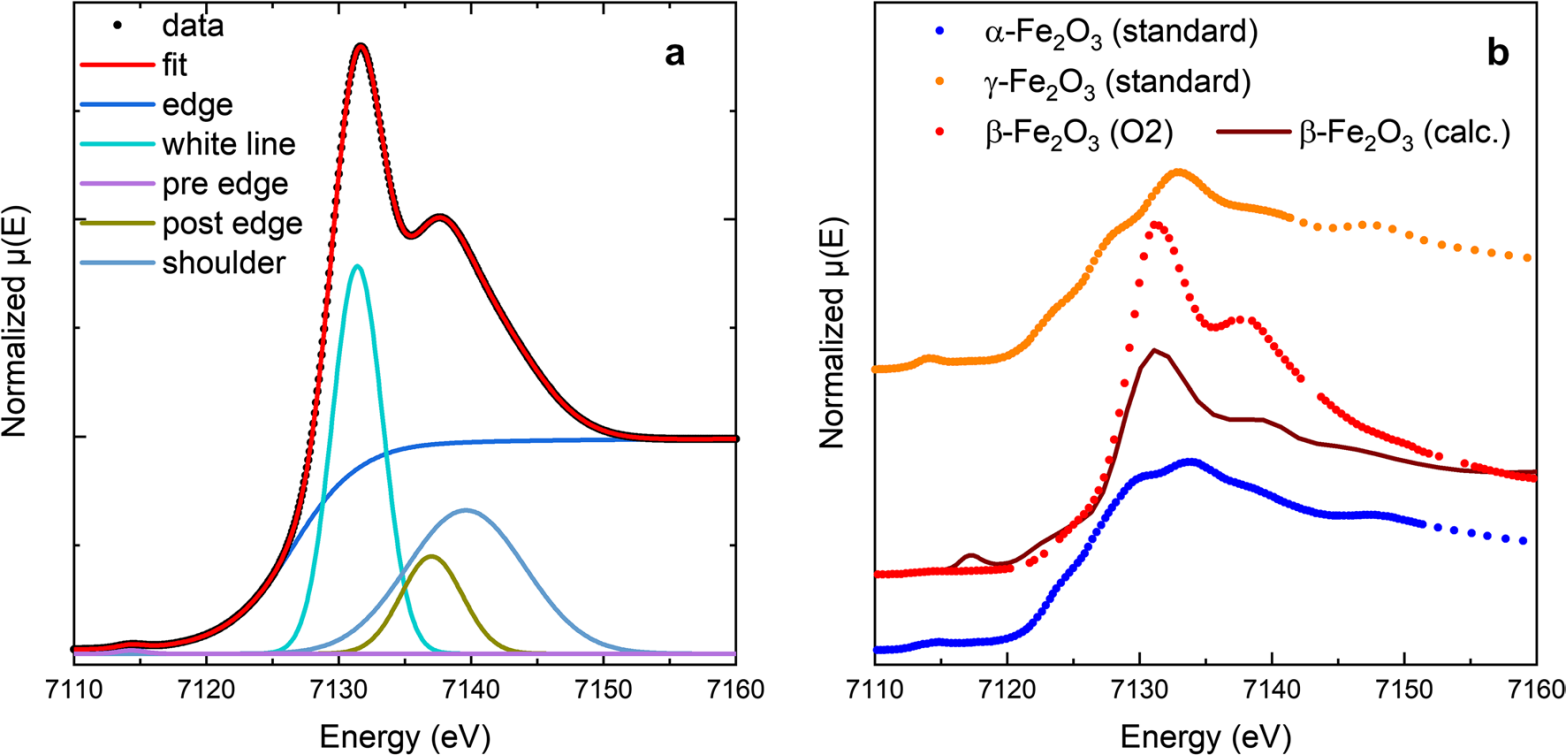
Experimental XANES with contributions to the semiempirical line shape analysis of individual features (**a**). Measured XANES spectra of β-Fe_2_O_3_ (sample O2) (red), α-, and γ-Fe_2_O_3_ standards (blue and orange) and corresponding FEFF simulation of β-Fe_2_O_3_ in dark red (**b**).

Qualitatively, the XANES spectra of the O2 sample is significantly different from the spectra for γ-Fe_2_O_3_ and α-Fe_2_O_3_ standards. The XANES spectra of iron (III) oxides display at least three characteristic features, pre-edge peaks at about 7115 eV, edge steps at about 7126 eV and white lines at about 7130 eV whose individual contributions together with the post-edge peak and shoulder are shown in Fig. 7a for sample O2. Results of the quantitative analysis are compiled in Table 4.

**Table 4:** XANES features of the β-Fe_2_O_3_ compared to α- and γ-Fe_2_O_3_ references.

Material	Pre-edge peak (eV)	Edge E0 (eV)	White line (eV)	Normalized white line intensity
β-Fe_2_O_3_	7115.8 (3)	7126.6 (3)	7131.6 (2)	2.74 (1)
α-Fe_2_O_3_	7114.9 (1)	7125.9 (2)	7133.6 (3)	1.49 (1)
γ-Fe_2_O_3_	7114.3 (2)	7125.7 (2)	7132.7 (2)	1.56 (1)

Measured XANES spectra together with the FEFF simulated spectrum for β-Fe_2_O_3_ are displayed in Fig. 7b. In case of pure nanocrystalline β-Fe_2_O_3_ (sample O2) the pre-edge peak is very broad and very low in intensity compared to the γ-Fe_2_O_3_ and α-Fe_2_O_3_ reference materials and the corresponding FEFF simulation. The edge energies, E0, are analyzed using a step function—a linear combination of arctangent and error function. The edge position for β-Fe_2_O_3_ (7126.6 (3) eV) is larger by about 0.7 and 0.9 eV compared to 7125.9 (2) eV for α-Fe_2_O_3_ and 7125.7 (2) eV for γ-Fe_2_O_3_, respectively. The intensity of the white line of β-Fe_2_O_3_ is substantially larger compared to the two other phases and the simulated spectrum considered. The energy of the white line of the synthesized β-Fe_2_O_3_ (7131.1 eV) on other hand is smaller than that of the investigated standards with 7133.6 eV and 7132.7 eV for α-Fe_2_O_3_ and γ-Fe_2_O_3_, respectively. The differences between β-Fe_2_O_3_ and the other polymorphs is larger compared to the difference between γ- and α-Fe_2_O_3_. In general, the energetic positions of spectral features in experimental data and FEFF simulations agree quite well, however the intensity is different. The use of an ideal crystal structure data for FEFF simulations does not take into account complex defect structures as expected for example for hollow particles in β-Fe_2_O_3_. Therefore, the simulated spectrum does not represent electronical structure completely.

**Figure 8:**
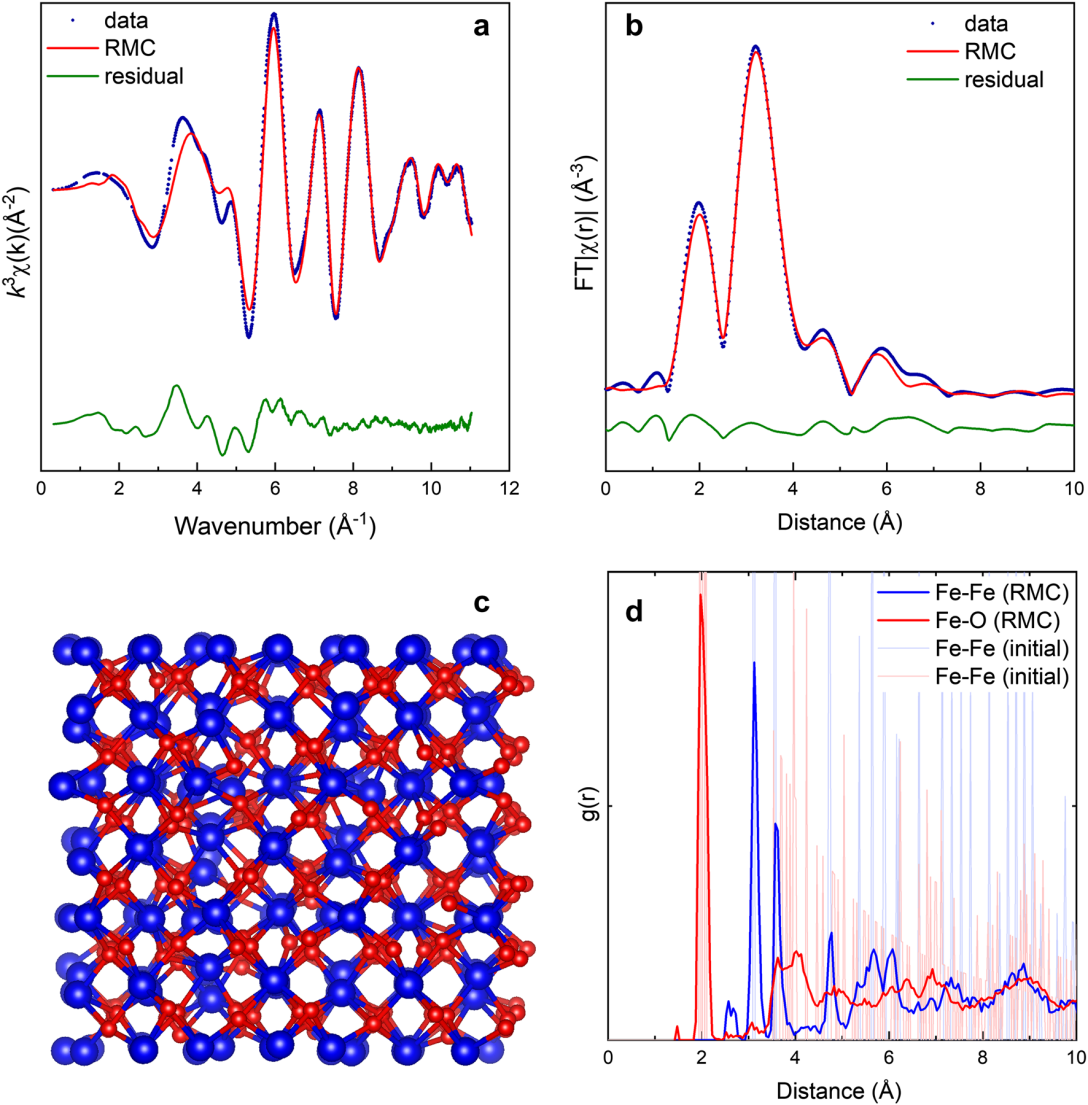
Results of analysis of EXAFS data for β-Fe_2_O_3_ (sample O2): k^3^-weighted raw EXAFS data of the measured sample and RMC-fit (**a**), corresponding phase corrected Fourier transformed spectrum (**b**), final atom configuration resulting from the RMC simulation (**c**) and corresponding partial PDF (**d**).

EXAFS data of sample O2 (pure β-Fe_2_O_3_) are analyzed using RMC simulations^29^ based on the crystallographic information obtained from Rietveld refinement of the XRD data. Figure 8a,b show the raw, unfiltered EXAFS data and the phase corrected Fourier transform with the RMC fit. The raw data and the Fourier transform are in good agreement with the RMC results. Deviations between experimental EXAFS data and RMC analysis (in k- and in r-space) are mainly due to insufficient background subtraction (low frequency signal, in r-space below about 1.8 Å) and multiple scattering not considered (high frequency signal, in r-space above about 4 Å). The final configuration of the β-Fe_2_O_3_ of the RMC simulation is shown in Fig. 8c. and the corresponding partial pair distribution functions (pPDF) in Fig. 8d. The results of the moment analysis of the Fe–O and Fe–Fe partial pair distribution function of the RMC refinement are compiled in Table 5 for the nearest and next nearest neighbors. The good agreement between EXAFS analysis and Rietveld refinement (Table 5) shows that long range order and local structure are consistent for nanocrystalline β-Fe_2_O_3_ with the exception of Fe–Fe pairs at 2.63 Å at distances significantly shorter than the next nearest neighbor coordination in β-Fe_2_O_3_ bixbyite. This may be due to iron interstitials or more likely due to a small remaining fraction of metallic iron as the distance is identical to the distance observed for bcc-iron clusters by Enz et al.^30^ a sample analogous to R3. These metallic clusters may be identified as intermediate precursor for the iron oxides in the CVS process described here. In β-Fe_2_O_3_ the Fe–O bond length of 2.03 Å is larger compared to γ-Fe_2_O_3_ or α-Fe_2_O_3_ with 1.92 Å and 1.93 Å respectively^32^ and^33^. The same is true for the Fe–Fe bond length with 3.09 Å compared to 2.85 Å in α-Fe_2_O_3_ and 2.99 Å in γ-Fe_2_O_3_^32^ and ^33^.

**Table 5:** RMC analysis results obtained for the nearest and next nearest neighbors by moment analysis of the pPDFs for β-Fe_2_O_3_ (sample O2): Coordination numbers (N), interatomic distances (R) and Debye–Waller factor σ^2^.

Atom pair		Range (Å)	β-Fe_2_O_3_ (RMC)	β-Fe_2_O_3_ (Rietveld)
Fe–O	N (–)		5.8 (14)	6.0
	R (Å)	1.8–2.8	2.03 (2)	2.03
	σ^2^ (Å^2^)		0.005 (2)	
Fe–Fe	N (–)		0.5 (1)	
	R (Å)	2.4–2.9	2.63 (2)	
	σ^2^(Å^2^)		0.005 (3)	
Fe–Fe	N (–)		6.2 (1)	6
	R (Å)	2.9–3.5	3.14 (5)	3.13
	σ^2^(Å^2^)		0.006 (3)	

For comparison, values based on the crystallographic data from Rietveld refinement of XRD data using the β-Fe_2_O_3_ bixbyite phase are included.

## Discussion

Possible microstructural models for the observed oxide materials are:

(i) Particles which are fully coalesced,
(ii) Particles with internal pores surrounded by a porous shell allowing access to nitrogen gas molecules and.
(iii) Particles containing internal pores (voids) and a dense shell.

These models are based on the following particle evolution scenario: precursor molecules are pyrolyzed forming metallic nuclei in a carbonaceous matrix, carbon is oxidized allowing metal particles to coalesce especially since the melting point of this ultrasmall metallic nanoparticles is very likely substantially lowered according to Eq. 2. The coalesced metal particles are oxidized from their surface and form an oxide shell and pores which are growing according to the Kirkendall effect with rising oxidation time. Finally, at long residence times in oxygen and at high temperatures sintering up to complete coalescence may occur and dense oxide particles are formed (microstructure i). Microstructure (ii) can probably not be avoided since the growing oxide shell with lattice constant different than the metal core cannot grow epitaxially on the shrinking metal core in all crystallographic directions. However, sintering of the polycrystalline shell may be able to generate a dense polycrystalline shell (microstructure iii).

Sample O1 consists of larger nanoparticles with emerging pores. With increasing oxidation time (O1 to O2) the number of pores and their diameter increases strongly, which can be attributed to Kirkendall growth. Subsequently, at higher oxidation time (O2 to O3), the average pore diameter is decreasing due to sintering. Sample O1 contains surprisingly the largest oxide crystals. The long residence time in reducing atmosphere preserves the small iron clusters in their carbon matrix which is then combusted and the resulting small iron oxide clusters coalesce rapidly to rather large γ-Fe_2_O_3_ and even α-Fe_2_O_3_ crystals.

In samples O2 and O3 we observe pores similar to those discovered by Lee et al.^4^. However, in contrast to Lee et al.^4^, we propose the Kirkendall effect as formation mechanism for hollow iron oxide nanoparticles, since iron acetylacetonate droplets at temperatures above 250 °C are very unlikely^34^ and because the evolution of porous nanoparticles is consistent with the Kirkendall mechanism.

From the increasing (crystallographic) density (Table 3) we propose an increasing thermodynamic stability from γ- to β- to α-Fe_2_O_3_. In case of sample O1 the shortest oxidation time is used. Therefore, the metallic precursor particles for the observed iron oxides experience the longest time for coalescence in the metallic state. Since coalescence is hindered by the carbon matrix and carbon interstitials a broader or even bimodal size distribution of primary particles may form. The critical size for the γ to α phase transformation is about 30 nm^2^. A fraction of 87 wt% γ-Fe_2_O_3_ particles of 29 nm diameter are just below while the remaining 13 wt% α-Fe_2_O_3_ of 32 nm diameter are above this threshold. Sample O2 consists of pure β-Fe_2_O_3_ with a crystallite size of about 20 nm. During the formation of sample O2 the longer oxidation time is sufficient to remove the carbon matrix earlier facilitating oxidation of smaller metallic precursor particles of narrower size distribution compared to sample O1 which results in the formation of phase pure β-Fe_2_O_3_ of 20 nm diameter. Although sample O3 experienced the longest oxidation time it contains only 62 wt.% of β-Fe_2_O_3_ with a crystallite size of about 20 nm and the remainder consists of γ-Fe_2_O_3_ 10 nm nanocrystals. The longest oxidation time for sample O3 causes the earliest oxidation of the iron metal particles when they are smallest. Coalescence, respectively sintering of the resulting oxide particles is probably slower compared to the metallic particles which is consistent with the smaller grain sizes observed in comparison to O2. Assuming there is also a critical size for the transformation from γ- to β-Fe_2_O_3_ only a fraction of particles is converted and remaining γ-Fe_2_O_3_ is observed. A critical size about 10 nm to 20 nm can be estimated from our results. According to our observations, the occurrence of the β-Fe_2_O_3_ phase is correlated to their shape—the appearance of the hollow spheres and large pores in the sample.

## Summary and conclusions

A double wall CVS reactor setup enables the reproducible synthesis of porous nanoparticles containing α-Fe_2_O_3_, β-Fe_2_O_3_ and γ-Fe_2_O_3_. Phase composition, pore and particle dimensions are tuned by varying oxidation time. A parameter window at intermediate oxidation time is identified for the facile gas phase synthesis of phase pure, hollow β-Fe_2_O_3_ nanoparticles. In general, the setup enables the decoupling of the formation of nanoparticles from a sequential reaction such as oxidation. Therefore, the presented setup may be used for the investigation of other residence- or reaction time dependent processes and allows the investigation of their kinetics.

## Data Availability

The datasets used and/or analyzed during the current study are available from the corresponding author on reasonable request.
